# Anti-Cancer Effects of an Optimised Combination of Ginsenoside Rg3 Epimers on Triple Negative Breast Cancer Models

**DOI:** 10.3390/ph14070633

**Published:** 2021-06-30

**Authors:** Maryam Nakhjavani, Eric Smith, Helen M. Palethorpe, Yoko Tomita, Kenny Yeo, Tim J. Price, Amanda R. Townsend, Jennifer E. Hardingham

**Affiliations:** 1 Molecular Oncology, Basil Hetzel Institute, The Queen Elizabeth Hospital, Woodville South, SA 5011, Australia; maryam.nakhjavani@adelaide.edu.au (M.N.); yoko.tomita@adelaide.edu.au (Y.T.); a1811332@student.adelaide.edu.au (K.Y.); jennifer.hardingham@adelaide.edu.au (J.E.H.); 2 Adelaide Medical School, University of Adelaide, Adelaide, SA 5005, Australia; timothy.price@sa.gov.au (T.J.P.); amanda.townsend@sa.gov.au (A.R.T.); 3 Centre for Cancer Biology, University of South Australia and SA Pathology, Adelaide, SA 5000, Australia; helen.palethorpe@unisa.edu.au; 4 Oncology Unit, The Queen Elizabeth Hospital, Woodville South, SA 5011, Australia

**Keywords:** ginsenoside Rg3, Epimer, triple negative breast cancer, metastasis, response surface methodology, nod *scid* gamma mice

## Abstract

Key problems of chemotherapies, as the mainstay of treatment for triple-negative breast cancer (TNBC), are toxicity and development of tumour resistance. Using response surface methodology, we previously optimised the combination of epimers of ginsenoside Rg3 (Rg3) for anti-angiogenic action. Here, we show that the optimised combination of 50 µM SRg3 and 25 µM RRg3 (C3), derived from an RSM model of migration of TNBC cell line MDA-MB-231, inhibited migration of MDA-MB-231 and HCC1143, in 2D and 3D migration assays (*p* < 0.0001). C3 inhibited mammosphere formation efficiency in both cell lines and decreased the CD44^+^ stem cell marker in the mammospheres. Molecular docking predicted that Rg3 epimers had a better binding score with IGF-1R than with EGFR, HER-2 or PDGFR, and predicted an mTOR inhibitory function of Rg3. C3 affected the signalling of AKT in MDA-MB-231 and HCC1143 mammospheres. In a mouse model of metastatic TNBC, an equivalent dose of C3 (23 mg/kg SRg3 + 11 mg/kg RRg3) or an escalated dose of 46 mg/kg SRg3 + 23 mg/kg RRg3 was administered to NSG mice bearing MDA-MB-231-Luc cells. Calliper and IVIS spectrum measurement of the primary and secondary tumour showed that the treatment shrunk the primary tumour and decreased the load of metastasis in mice. In conclusion, this combination of Rg3 epimers showed promising results as a potential treatment option for TNBC patients.

## 1. Introduction

Triple-negative breast cancer (TNBC) is a subtype of breast cancer for which limited targeted therapy is available. Chemotherapy is the mainstay of the treatment for TNBC. Administration of various chemotherapies can be limited by toxicities and the development of tumour resistance. Despite using selected chemotherapy and neoadjuvant chemotherapy regimens, these patients have a higher rate of developing visceral metastases [1] with pathological complete response of 33% [2].

Cancer cells undergo epithelial to mesenchymal transition (EMT), and acquire cancer stem cell characteristics, one of the mechanisms by which cells become resistant to chemotherapies [3]. Different subtypes and stages of breast cancer have their unique expression of stem cell markers such as CD44, CD24 and aldehyde dehydrogenase (ALDH) [4] and the search for drugs which reduce cancer stemness is an essential investigation in drug discovery and development programs. Phosphatidylinositol 3-kinase/protein kinase B/mammalian target of rapamycin (PI3K/AKT/mTOR) pathway is one of the several self-renewal pathways existing in breast cancer cells [5,6,7] and inhibitors of this pathway may reduce ‘stemness’ of cancer cells. Targeting this pathway is therefore important in overcoming drug resistance, cell survival and metastasis in this cancer [8]. Several clinical studies are ongoing to evaluate the efficacy of the inhibitors of this pathway in TNBC patients (reviewed in [5]).

Ginsenoside Rg3 (Rg3) is a member of the ginsenosides family of molecules extracted from *Panax ginseng*. The molecule has two epimers, 20(S)-Rg3 (SRg3) and 20(R)-Rg3 (RRg3). We have previously discussed the potential of these molecules as a treatment for metastatic breast cancer [9,10]. In addition, studies showed that Rg3 decreased the activation of PI3K/AKT pathway in several tumour models such as leukaemia, ovarian and lung carcinoma (reviewed in [9]). Based on this background, Rg3 might be a potential candidate for the treatment of metastatic TNBC (mTNBC). We previously showed that the epimers of Rg3 have stereoselectivity in their anti-cancer actions [11], as only SRg3 inhibited the proliferation of MDA-MB-231 (100 µM) and blocked aquaporin 1 (AQP1) water channel (50 µM), which plays important roles in the proliferation, migration, and invasion of cancer cells and in angiogenesis. Furthermore, it was only RRg3 that inhibited the invasion of MDA-MB-231 cells. SRg3 and RRg3 inhibited the migration of TNBC cells in wound closure and transwell migration assays with different manners [11]. Based on these findings, SRg3 and RRg3 could be considered as two different drugs. Therefore, we determined the optimal concentration of SRg3 and RRg3 using response surface methodology (RSM) modelling. RSM is one of the well-established methods in pharmaceutical science and in studying drug combinations, which is especially important and helpful in cancer treatment studies [12,13,14]. The optimisation, which was based on the anti-angiogenic effects of the molecules [15], showed that a combination of 50 µM SRg3 + 25 µM RRg3 had the best efficacy in inhibition of loop formation and cell migration of endothelial cells. This treatment induced cell death and cell cycle arrest in human and murine endothelial cell lines, and decreased the expression of vascular endothelial growth factor (VEGF), AQP1 and the phosphorylation of several proteins downstream of activation of AKT.

The current study aimed at investigating the efficacy of this combination on TNBC cell lines and in a murine model of mTNBC. The optimised combination of both molecules was assessed using RSM to confirm that the combination is effective in inhibiting TNBC cell migration. Next, the efficacy of the treatment was studied on mammospheres, as a more relevant model of human breast tumour and the effects of the treatment on stem cell markers and PI3K/AKT signalling pathway was investigated. Lastly, the in vivo efficacy of the treatment was studied in an orthotopic model of murine mTNBC. A diagram depicting the steps undertaken in this study is shown in Supplementary Figure S1.

## 2. Results

### 2.1. Response Surface Methodology Modelling

**Figure 1:**
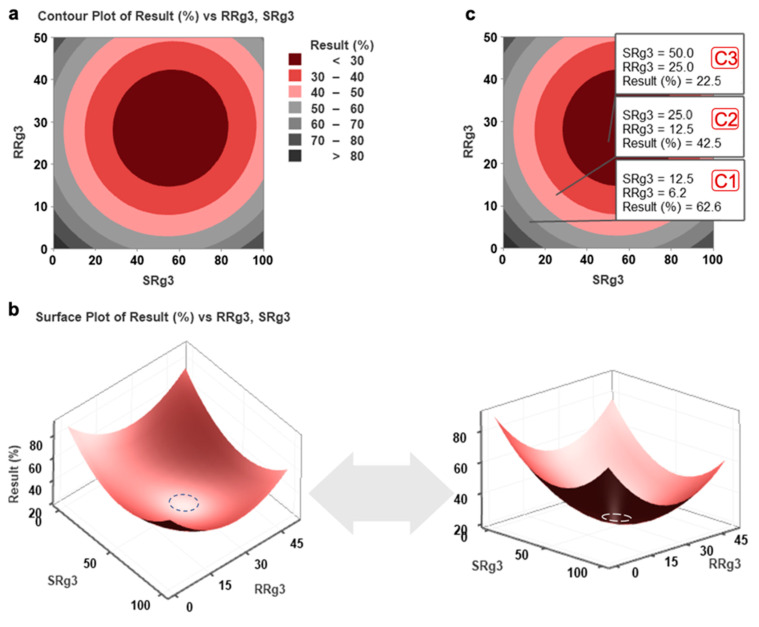
The results of response surface methodology (RSM) on the migration of MDA-MB-231 cell line. The calculated (**a**) contour plot and (**b**) 3D surface plots (viewed from two angles) for cell migration based on the response surface methodology model developed to optimise and confirm the efficacy of SRg3 + RRg3 drugs in combination in MDA-MB-231 cell line. Dashed circles show the optimised minimal response area. (**c**) The predicted cell migration (%) with different combinations of SRg3 and RRg3. Results (%) is the percentage of expected migration.

To identify the optimal combination concentration of SRg3 and RRg3 that inhibited TNBC cell line migration in vitro, we performed response surface methodology (RSM) modelling (Figure 1). The RSM contour plot (Figure 1a) shows the predicted effect of different concentrations of SRg3 and RRg3 on MDA-MB-231 migration. Figure 1b shows the data represented as a 3D surface plot, and the optimal combination concentration is highlighted with a dashed-line circle. The RSM modelling suggest that 50 µM SRg3 and 25 µM RRg3 (C3) was the optimal in vitro combination, inhibiting MDA-MB-231 migration to 22.5%.

RSM, like other methods of drug interaction studies, is cell line-dependent. Therefore, the results obtained in this method might not necessarily apply to other cell lines. In addition, we performed RSM for the migration of HCC1143 (Supplementary Figure S2). This produced a different model, which predicted that C3 inhibited migration to 10%. Based on these findings, C3 was used for subsequent experiments.

### 2.2. Rg3 Inhibits Migration but Not Proliferation in TNBC Cell Lines

**Figure 2:**
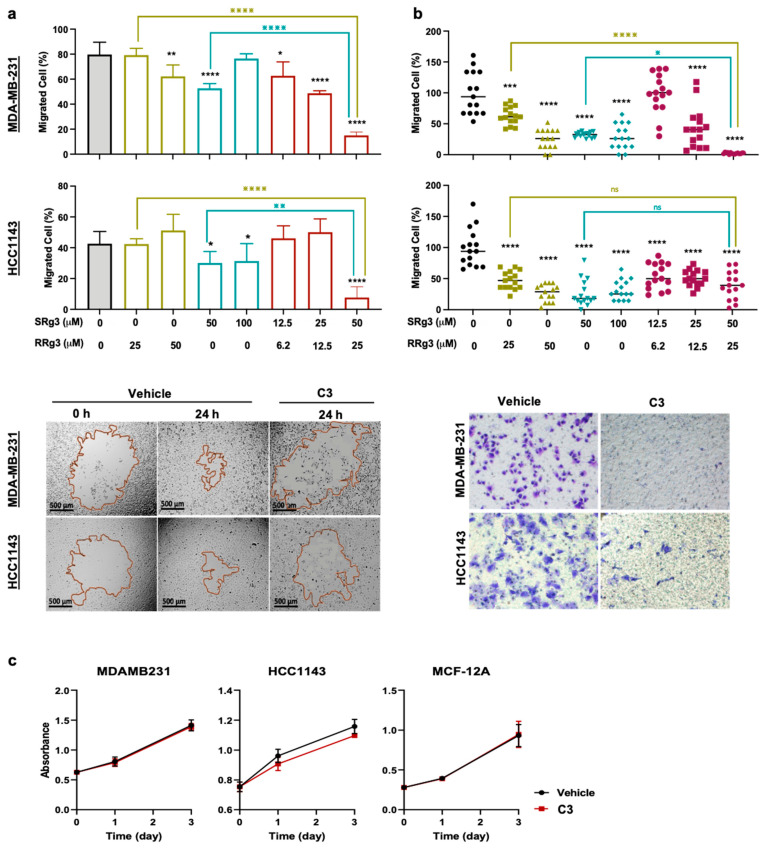
Effects of SRg3, RRg3 and the combination of both in (**a**) circular scratch migration and (**b**) transwell migration assays on MDA-MB-231 and HCC1143. Cells were treated for 3 days. Datapoints show mean ± standard deviation (SD) of (**a**) 6 and (**b**) 3 replicates. (**c**) Anti-proliferative effect of C3 (50 µM SRg3 + 25 µM RRg3) on TNBC cell lines (MDA-MB-231 and HCC1143) and a normal breast cell line (MCF-12A), using crystal violet assay (mean ± SD). Significant differences between the vehicle versus treatments (black-coloured asterisk), 25 µM RRg3 versus C3 (green asterisk) and 50 µM SRg3 versus C3 (teal asterisk) are shown at * *p* < 0.05, ** *p* < 0.01, *** *p* < 0.001 and **** *p* < 0.0001.

To test and show the efficacy of C3, the effect of individual or combinations of Rg3 epimers on migration of TNBC cells was tested in a circular scratch-wound (2D) and a transwell (3D) migration assay (Figure 2).

After a three-day pre-treatment with individual or combinations of Rg3 epimers, MDA-MB-231, compared to HCC1143, showed more sensitivity to the anti-migratory effects of Rg3 only in the 2D assay. In MDA-MB-231, each of the three tested combinations, C3, C2 and C1, significantly inhibited cell migration to 15%, 38% and 52% of vehicle control, respectively (Figure 2a). This was similar to the predicted RSM model of <30%, 40–50% and 60–70% for C3, C2 and C1, respectively (Figure 1b). It is noteworthy that with SRg3, a concentration-dependent response is not observed and inhibition of migration at 100 µM is less than at 50 µM. This might be partly due to the cytotoxic effect of SRg3 on MDA-MB-231 at this concentration [11] and a potential U-shaped dose-response curve for SRg3 (reviewed in [10]), which requires further investigation. In HCC1143, 50 and 100 µM SRg3 inhibited cell migration by about 30% (*p* < 0.05) and among the combinations, only C3 significantly inhibited the 2D migration of cells (*p* < 0.0001) (Figure 2a). These results also indicated that the average cell migration with C1 and C2 was about 62% and 48%, respectively, which fall into the appropriate regions predicted by RSM, being 60–70% for C1 and 40–50% for C2 (Figure 1b). C3 inhibited cell migration by about 85% and 92% in MDA-MB-231 and HCC1143 cell lines, respectively. As shown in Figure 2a, in both cell lines, the effect of C3 on cell migration was significantly more than either of the single epimers.

The transwell assay showed that in both cell lines, single epimers significantly inhibited cell migration (*p* ≤ 0.0002) (Figure 2b). All three combinations in HCC1143 and C2 and C3 in MDA-MB-231 showed efficacy in inhibition of 3D migration of cells (*p* < 0.0001). In MDA-MB-231, C3 showed the highest efficacy by almost completely inhibiting cell migration across the membrane (*p* < 0.0001). This effect was significantly more than the effect of either of the epimers as single agents. However, in HCC1143, the effect of combinations was not significantly different from single molecules (Figure 2b).

Figure 2c shows the anti-proliferative effects of C3 on the TNBC cell lines and a normal breast epithelial cell line. As shown in this figure, this treatment did not inhibit the proliferation of these cell lines.

### 2.3. Rg3 Decreases Mammosphere Formation Efficiency (MFE) in TNBC 3D Models, via Decreasing ‘Stemness’ of the Cells

**Figure 3:**
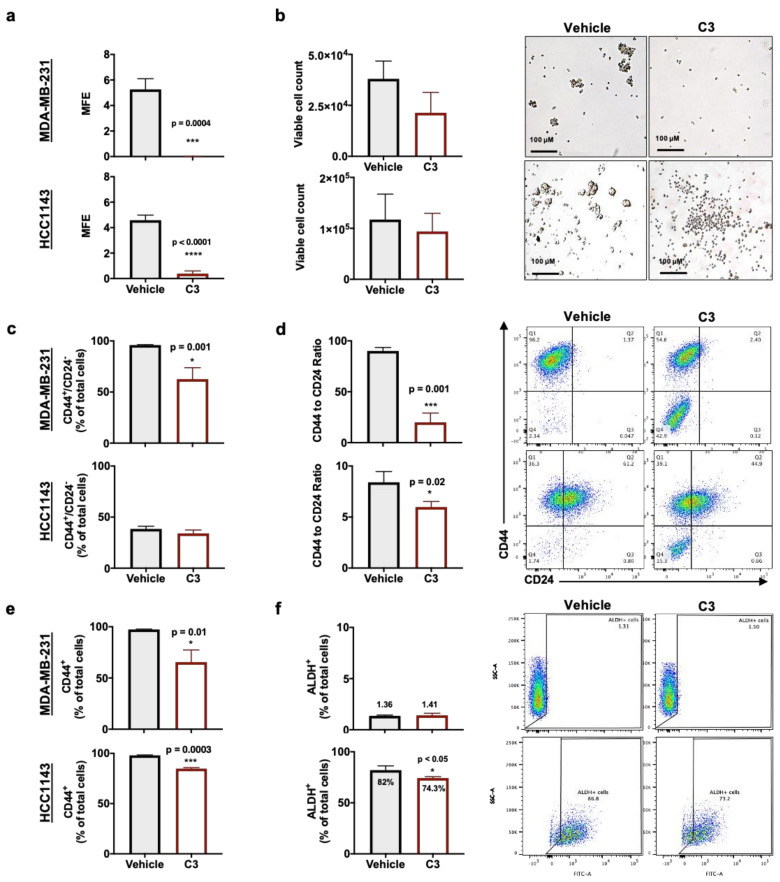
MDA-MB-231 and HCC1143 grown as mammospheres and exposed to C3 (50 µM SRg3 + 25 µM RRg3) for 4 days. (**a**) Mammosphere formation efficiency (MFE). Mechanisms of inhibition of MFE was studied by testing (**b**) viability of the mammospheres, expression of (**c**) CD44^+^/CD24^−^ phenotype, (**d**) ratio of CD44 and CD24 expressing cells, (**e**) CD44^+^ expression and (**f**) expression of ALDH. Each datapoint represents mean ± SD of three replicates. Statistical comparisons are between the treated and vehicle groups, * *p* < 0.05, *** *p* < 0.001 and **** *p* < 0.0001.

Breast cancer stem cells (BCSC) play important roles in disease progression, metastasis and recurrence. Therefore, following showing the efficacy of C3, we investigated the efficacy of C3 to alter BCSC self-renewal using a three-dimensional (3D) mammosphere formation efficiency (MFE) assay [16,17]. Treatment with C3 significantly reduced the MFE of MDA-MB-231 (*p* = 0.0003) and HCC1143 (*p* < 0.0001) (Figure 3a). Treatment with C3 did not significantly decrease the viable cell count (Figure 3b). There was no evidence to suggest that C3 treatment induced apoptosis (Supplementary Figure S3a) or cell cycle arrest (Supplementary Figure S3b). Taken together, these results suggest that the reduction in MFE was not due to a reduction in cell viability.

To determine if the reduction in MFE was due to a reduction in the number of stem cells, we evaluated the BCSC markers CD44^+^/CD24^−/low^ (Figure 3c,d) and ALDH (Figure 3e) [4]. It was shown that a high ratio of cells expressing CD44 to CD24 was correlated with strong tumorigenicity of breast cancer and ALDH was correlated with the metastatic capacity of the tumour. These markers were correlated with breast cancer malignancy [4]. The CD44^+^/CD24^−/low^ phenotype was also correlated with poorer prognosis in TNBC patients and together with ALDH^+^ are expressed in axillary lymph node (aLN) metastasis [4,18].

MDA-MB-231 and HCC1143 cell lines have mesenchymal and epithelial characteristics, respectively, which is also reflected in the expression of CD44^+^/CD24^−/low^ phenotype in mammospheres. Figure 3c,d show that compared to HCC1143, MDA-MB-231 had higher expression of CD44^+^/CD24^−^ phenotype as mammospheres. Exposure to C3 decreased the expression of CD44^+^/CD24^−^ phenotype in MDA-MB-231 mammospheres by 35% (*p* = 0.001) (Figure 3c). MDA-MB-231 showed a more significant decrease in the ratio of cells expressing CD44 to CD24 (*p* = 0.001); a double negative subpopulation was also observed (Figure 3c,d). Likewise, with HCC1143 mammospheres, upon exposure to C3, a double-negative subpopulation of cells was created and the ratio of cells expressing CD44 to CD24 was significantly decreased (*p* = 0.02) (Figure 3d). In both mammospheres, expression of CD44 was significantly decreased (Figure 3e). MDA-MB-231 cells express low levels of ALDH [19] and that expression was not altered by C3. Expression of ALDH in HCC1143 was only slightly decreased by C3 treatment (*p* < 0.05) (Figure 3e). Together, these findings suggest that C3 treatment reduced the proportion of BCSC.

### 2.4. Effect of Rg3 on Akt/mTOR Signalling

To study other contributing mechanisms of C3 in inhibition of MFE, role of AKT/mTOR signalling in mammospheres was investigated. This signalling is commonly initiated by the activated receptor tyrosine kinases (RTKs). Therefore, using in silico molecular docking, we screened for potential RTK targets and, using phospho-protein array, investigated the effect of C3 on AKT/mTOR signalling in mammospheres.

**Figure 4:**
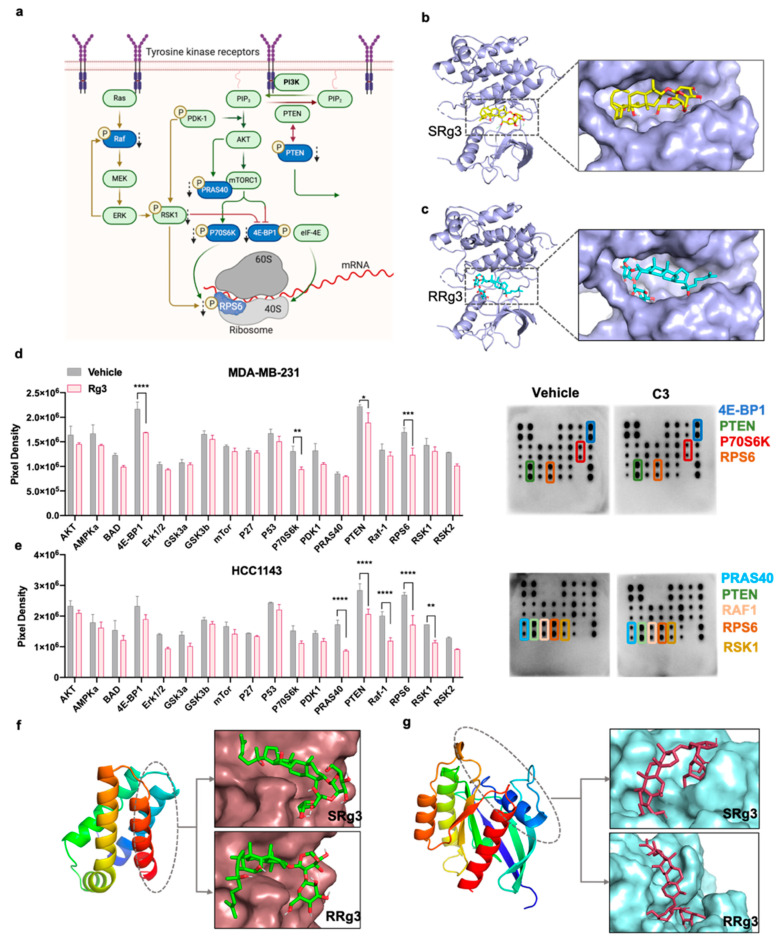
(**a**) An illustration of signalling of PI3K/AKT/mTOR and Ras/Raf/MEK/ERK signalling pathways (created with BioRender.com, January 2021). Dashed black arrows show where Rg3 is affecting, and affected proteins are shown in blue. Interaction of (**b**) SRg3 and (**c**) RRg3 with ATP-binding pocket of IGF-1R. The effects of C3 (50 µM SRg3 + 25 µM RRg3) on the expression of phosphorylated proteins downstream of activation of AKT signalling in (**d**) MDA-MB-231 and (**e**) HCC1143 mammospheres. The data are the mean ± SD of two replicates (**left**), derived from the presented dot blots (**right**). Statistical comparisons are between the treated and vehicle groups, * *p* < 0.05, ** *p* < 0.01, *** *p* < 0.001 and **** *p* < 0.0001, (**f**) FKBP12 rapamycin-binding site of mTOR binding to SRg3 and RRg3 and (**g**) Rheb protein binding to SRg3 and RRg3.

RTKs such as epidermal growth factor receptor (EGFR) and insulin-like growth factor-1 receptor (IGF-1R) are overexpressed in TNBC, are considered to be prognostic and predictive markers [20,21,22] and are potential targets for treatment. Activation of RTKs triggers multiple signalling pathways which guarantees cell cycle progression, proliferation, survival, migration and angiogenesis (Figure 4a). We had previously shown that Rg3 interacts with VEGFR2, in silico and in vitro, and functions as an allosteric modulator of the receptor [15]. In this study, we screened the interaction of Rg3 with some other RTKs.

Table 1 shows the binding scores of Rg3 epimers with EGFR, human epidermal growth factor receptor-2 (HER-2), IGF-1R and platelet-derived growth factor receptor (PDGFR). The interaction sites of SRg3 and RRg3 with amino acids within IGF-1R, FRB and Rheb, represented from the best binding score out of 10, 9 and 9 conformational positions, respectively, are shown in Supplementary Table S1 and Supplementary Figure S4. The binding scores were compared with those of two known tyrosine kinase inhibitors (TKIs), sorafenib and lenvatinib.

As shown in Table 1, both TKIs are predicted to have good binding scores with the receptors. With Rg3 epimers, the best scores belonged to the interaction of SRg3 and RRg3 with IGF-1R, being −8 and −7.5 kJ/mol, respectively, each among 10 different conformational positions. Figure 4b,c show the interaction of SRg3 and RRg3 with ATP- binding pocket of IGF-1R, respectively. As shown in these Figures, Rg3 molecules are predicted to be well-placed in the ATP-binding pocket of the receptor.

Following activation of an RTK, different downstream signalling pathways might be activated. PI3K/AKT and Ras/Raf/MEK/ERK are two such pathways in crosstalk. PI3K phosphorylates and activates AKT. AKT has several targets, the action of which leads to cell survival, migration, proliferation and angiogenesis. One of the important targets of AKT is mammalian target of rapamycin complex 1 (mTORC1), which is sensitive to and regulated by several signals including nutrients and growth factors [23]. Two key substrates of mTORC1 are eukaryotic translation initiation factor 4E (eIF4E)-binding protein 1 (4E-BP1) and P70 ribosomal protein S6 kinase (P70S6K). They are crucial factors in translation initiation that predominantly mediate the translational functions of mTORC1. mTORC1, via phosphorylation of 4E-BP1, inactivates this protein, releasing p-4E-BP1 from eIF-4E. Activated eIF-4E then initiates translation (Figure 4a). mTORC1 also phosphorylates and activates P70S6K. Activated P70S6K phosphorylates and activates ribosomal protein S6 (RPS6), a component of S40 ribosomal subunit, which plays an important role in global translation and cell growth and glucose homeostasis (Figure 4a) [24].

The effect of C3 on the phosphorylation of proteins downstream of activation of AKT on two TNBC mammosphere models was tested using AKT Pathway Phosphorylation Array and the result is shown in Figure 4d,e. Mammospheres, originated from two different TNBC cell lines with mesenchymal (MDA-MB-231) or epithelial (HCC1143) characteristics, reacted differently to C3.

In MDA-MB-231, the expression of phosphorylated forms of 4E-BP1 (*p* < 0.0001), RPS6 (*p* = 0.0001), P70S6K (*p* = 0.0032) and phosphatase and tensin homolog (PTEN) (*p* = 0.0109) were significantly decreased (Figure 4d). PTEN is a tumour suppressor gene, an inhibitor of the activation of PI3K and its subsequent signalling (Figure 4a). In this study, phosphorylation of Ser380, which inactivates PTEN was measured. It was shown that C3 significantly decreased the levels of inactive phosphorylated PTEN (*p* = 0.0109), which would improve the tumour suppressor function. In MDA-MB-231, C3 also reduced mTOR-initiated ribosomal translation via decreased phosphorylation of 4E-BP1, P70S6K and RPS6.

Similar to MDA-MB-231, C3 treatment of HCC1143 mammospheres reduced the levels of the phosphorylated forms of PTEN (*p* < 0.0001) and RPS6 (*p* < 0.0001). In addition, the expression of phosphorylated forms of proline-rich AKT substrate of 40 kDa (PRAS40) (*p* < 0.0001), Raf-1 (*p* < 0.0001) and ribosomal S6 kinase (RSK1) (*p* = 0.0038) were also decreased (Figure 4e). PRAS40 is a substrate of AKT and a component and substrate of mTORC1 (Figure 4a). AKT-mediated phosphorylation of PRAS40 at Thr246, studied in this assay, inactivates PRAS40 in a way that mTORC1 restores its function [23]. Hence, decreased phosphorylation of PRAS40 decreases the activation of mTORC1.

RSK1 is a member of the family of 90 kDa RSKs. RSKs are activated downstream of activation of Ras/Raf/MEK/ERK pathway. The signalling of Ras/Raf/MEK/ERK plays roles in cell proliferation, survival, migration and angiogenesis. It is in interaction with the signalling of the AKT pathway. Upon activation, RSKs play roles in regulating several cellular functions, including cell proliferation, survival and motility [25], via interaction with phosphorylation of 4E-BP1 and RPS6 [26]. Raf-1 is a proto-oncogene and a serine/threonine protein kinase that is activated downstream of activation of Ras, leading to activation of MEK and ERK. Phosphorylation at SER-301 of Raf-1, studied in this assay, is a result of ERK-mediated feedback phosphorylation of Raf-1 [27].

Considering the changes in the phosphorylation of proteins downstream of activation of mTOR, we performed a molecular docking to find out if Rg3 epimers have any good binding with mTORC1. In this preliminary screening, we tested two sites of the complex, the FRB and Rheb. The FRB site is one of the functional domains of mTOR kinase, where rapamycin binds. At this site, SRg3 and RRg3 have binding score of −7 and −7.2 kJ/mol, respectively, which are comparable to the binding score of rapamycin, −7.6 kJ/mol (Table 1). Furthermore, SRg3 and RRg3 had binding scores of −8.0 kJ/mol with Rheb. Rheb is a GTP-binding protein that, upon insulin and growth factor stimulation, binds to and activates mTORC1. Molecular docking showed that Rg3 has a good binding score with Rheb and could thus decrease the signalling of mTOR axis, the inhibition of which is important in cancer treatment [28,29].

### 2.5. In Vivo Evaluation of the Efficacy of Rg3 Combo

Finally, we evaluated the in vivo efficacy of Rg3 combinations. Two in vivo studies were conducted where MDA-MB-231 mammospheres were inoculated into mouse mammary fat pad. In the first study, the efficacy of C3 equivalent dose, combo A (23 mg/kg SRg3 + 11.5 mg/kg RRg3), was studied, and in the second study, a dose-escalation study, the efficacy of combo B (46 mg/kg SRg3 + 23 mg/kg RRg3) was evaluated.

**Figure 5:**
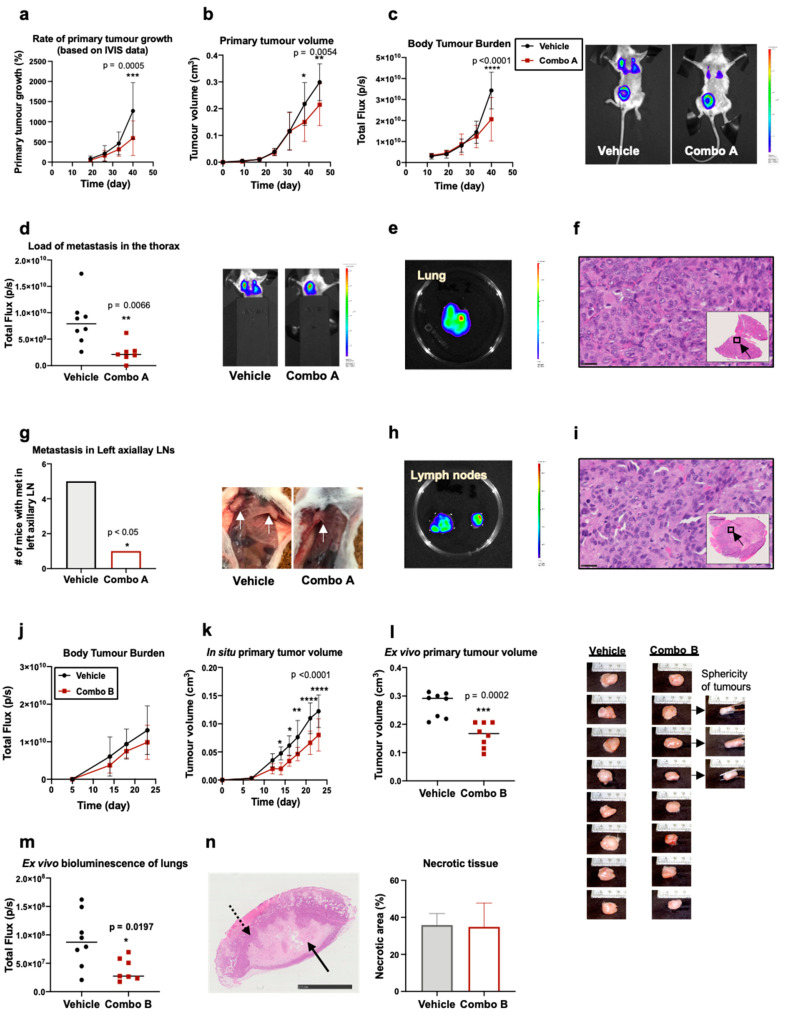
Outcomes of administration of combo A and B to a metastatic model of TNBC in Nod *scid* gamma (NSG) mice. MDA-MB-231 cells were grown as mammospheres and inoculated in the mammary fat pads of NSG mice. Administration of combo A started on day 12 and (**a**) decreased the rate of primary tumour growth measured with IVIS spectra, (**b**) primary tumour volume measured with a calliper, (**c**) body burden of the tumour, (**d**) the load of metastasis in the thorax and (**g**) number of enlarged left axillary lymph nodes, indicated with white arrows. (**e**,**h**) Show representative ex vivo IVIS images of lung and lymph nodes and (**f**,**i**) are the results of histology on lung and lymph nodes, respectively, showing tumour cells in the tissues. Black arrows show the region in the tissue that is magnified. The scale bar shows 25 µm. In a dose escalation study, administration of combo B to mice started on day 5 and (**j**) decreased, though not significantly, the average body burden of the tumour, (**k**) primary tumour volume measured with a calliper in situ or (**l**) ex vivo. (**m**) The treatment also significantly decreased the load of tumour in the lungs of mice, (**n**) there were no significant differences between the groups in terms of necrotic or viable area; black arrow shows the necrotic area and dashed arrow shows the proliferative region and the scale bar shown 2.5 mm. Each treatment group included 8 mice. All IVIS scales show count values between 600–60000. The data presented in (**a**–**c**,**j**–**n**) are mean ± SD. * *p* < 0.05, ** *p* < 0.01, *** *p* < 0.001 and **** *p* < 0.0001.

With combo A, weekly IVIS imaging demonstrated that the treatment significantly decreased the growth rate of the primary tumour (*p* = 0.0005) (Figure 5a). As shown in this Figure 5a, in both groups, tumours are growing bigger over time, but in the vehicle group, the average rate of tumour group is faster than the treatment group. Therefore, combo A could significantly reduce the rate of the primary tumour growth. This result was confirmed with calliper measurements (Figure 5b). This confirms that in both groups, tumour volume is increasing. However, the efficacy of the drug was observed after 30 days of treatment, when the average tumour volume was increasing with a lower slop in the treatment group. This might indicate that the treatment requires time to show efficacy in vivo. We can conclude that with adjusting the dosing frequency to once a day or twice a day, a better efficacy profile could be anticipated. IVIS imaging also showed that this treatment significantly decreased the total body tumour burden (*p* < 0.0001) (Figure 5c), which again followed a similar pattern as the primary tumour growth. On the last day of the study, IVIS imaging on the thorax area showed that combo A significantly decreased thoracic tumour load compared to the treatment group (*p* = 0.0066) (Figure 5d). Lung metastases were confirmed with ex vivo imaging (Figure 5e) and H&E staining (Figure 5f). Furthermore, treatment with combo A significantly reduced the number of metastatic aLNs (Figure 5g). Since the primary tumour was inoculated to the right mammary fat pad (MFP), it was closer to the right aLN than the left one. Therefore, tumour cells could easily migrate to the right aLN and get to the lung, and hence, mice in both groups had an enlarged ipsilateral (right) aLNs. Five of eight mice in the vehicle group showed an enlarged contralateral (left) aLN, whilst only one mouse in the treatment group had an affected left aLN. This result shows that combo A decreased the load of metastasis in LNs or reduced the rate metastasis to aLNs. Ex vivo imaging (Figure 5h) and H&E staining (Figure 5i) confirmed the presence of tumours in the metastatic aLNs.

Next, in a dose escalation study, we doubled the doses to 46 mg/kg SRg3 and 23 mg/kg RRg3 (combo B). Administration of a higher dose of Rg3 to mice caused a lessening trend in the average total flux of the whole body (Figure 5j), which started as early as the second dose. However, this decrease in total body flux was not statistically significant. The escalated dose also significantly decreased the size of the primary tumour. Between days 12 and 14, the average tumour volume did not change in the treatment group, while in the vehicle group, the average tumour size was increasing. This could indicate that doubling the dose induced a faster tumour response in this mouse model. A significant difference between the treatment and the vehicle group was noticed as early as day 14 (*p* = 0.01). Tumour volume measurements on days 16 (*p* = 0.01), 18 (*p* = 0.0056), 21 (*p* < 0.0001) and 23 (*p* < 0.0001) confirmed the decreased size in the treatment group (Figure 5k). Ex vivo measurement confirmed that the treatment significantly decreased the primary tumour volume (*p* = 0.0002) (Figure 5l). It was also noticed that most of the tumours had a spherical shape, while three of the tumours in the treatment group had not grown in-depth and did not have a spherical structure (Figure 5l). Ex vivo imaging of the excised lungs also showed a significant decrease in the bioluminescence photons detected in the treatment group lungs (*p* = 0.0197) (Figure 5m). Furthermore, there were no significant differences between the groups in terms of necrotic or proliferative areas (Figure 5n). In these two studies, no signs or symptoms of drug toxicity were observed. There were no significant differences between the two groups in terms of weight loss, fur condition, posture and behaviour (Supplementary Figure S5).

## 3. Discussion

The objective of this research was to study and introduce a plausible treatment option for TNBC and mTNBC. In our previous studies, we showed that epimers of Rg3 have stereoselective activities [11] and then using an RSM model, the combination of these two epimers was optimised for its anti-angiogenic effects in vitro [15]. In the current study, first, the validity of the optimised combination was tested in MDA-MB-231, and it was shown that the combination (C3) exerted a high anti-migration effect in the circular scratch migration assay. The validity of the RSM model was also tested with two other combinations (C1 and C2). In both migration assays, C3 showed high efficacy in inhibiting cell migration of both TNBC cell lines, while it had no anti-proliferative effect on them. Then, the efficacy of C3 was tested in mammospheres, which better mimic the in vivo drug responses of human breast tumour. Formation of mammosphere is related to ‘stemness’ of the cancer cells [30]. The treatment significantly inhibited MFE. As tested in this study, one contributing reason for the decreased MFE could be the decreased ‘stemness’ of the cells.

In this study, combined expression of CD44 and CD24 were studied. CD44 and CD24 are markers that are more expressed on progenitor-like cells and more differentiated cells, respectively. In breast cancer cells with CD44^+^ phenotype, genes that are involved in cell motility and angiogenesis were highly expressed, and the cells were more mesenchymal, motile and predominately oestrogen receptor-negative. In breast cancer patients, the CD44^+^/CD24^−^ phenotype is related to triple negativity and unfavourable prognosis [31], worse clinical behaviour [32] and distant metastasis [33]. These cells expressed higher levels of pro-invasive genes and had highly invasive properties [34]. Studies also showed that downregulation of CD44 reduced doxorubicin resistance of CD44^+^/CD24^−^ breast cancer cells [35]. It was previously shown that 50 µM SRg3 decreased the CD44^+^/CD24^−^ subpopulation in MDA-MB-231 [36]. In our study, RRg3 was added as a co-treatment and the combination caused a double negative subpopulation of cells. In addition, it was previously reported that Rg3 inhibited EMT via inactivating P38 MAPK and Smad2 increased expression of E-cadherin and Snail and decreased expression of vimentin (reviewed in [9]). Decreased expression of CD44 might be another contributing factor to this phenomenon, because CD44 also plays roles in EMT [37].

The mechanism by which expression of these stem cell markers is decreased in these cells might be via inhibition of the PI3K/AKT pathway. It has been shown that inhibitors of this signalling pathway, such as quercetin [7] and BEZ235 (dactolisib) [38], decreased breast cancer ‘stemness’ via decreased expression of CD44^+^/CD24^−^ phenotype. Previously, Rg3 was shown to affect the PI3K/AKT signalling and decreased the activation of Akt, mTOR, GSK-3β, 4E-BP1, Src and P70S6K (reviewed in [9]). In the present study, it was shown that C3 affects AKT/mTOR signalling in both TNBC cell lines tested. Rg3 was also predicted to have a good binding score with IGF-1R and could be a blocker of mTOR activation via blocking the interaction site of Rheb with mTOR. mTOR regulates several cellular functions including cell adhesion, changes in extracellular matrix and migration [39]. C3 may have exerted its anti-cancer actions by affecting the regulatory function of mTOR.

We previously showed that in endothelial cells, C3 was an inhibitor of AKT signalling, with the highest efficacy on p-mTOR and its substrates, p-4E-BP1 and p-P70S6K. Consistent with those results, in MDA-MB-231, both of these proteins were affected. In addition, 4E-BP1 plays a role in mTOR-initiated translation of factors involved in metastasis such as matrix metaloproteinase-9, fibroblast growth factor, vascular endothelial growth factor, C-MYC and cyclin D1 [39]. Along with these roles, 4E-BP1 was introduced as an oncogene in breast cancer [40], playing a role in the proliferation of breast cancer cells [41]. Furthermore, mTORC1/4E-BP1 regulated neural stem cell renewal capacity [42], a pathway which might also play a role in stem cell renewal in breast cancer and needs further investigation.

p-P70S6K plays roles in cell growth and cell cycle progression, and is also identified as an important factor in breast cancer survival and predicting response to treatment [43]. This protein may also play roles in metastasis, as overexpression of p-P70S6K was linked to metastasis in gastric carcinoma [44]. It is also responsible for the phosphorylation of protein S6. RPS6 is involved in the regulation of cell size and glucose homeostasis [24]. Hyperphosphorylation of this protein via AKT/mTOR/p70S6K pathway was relevant to the progression of non-small cell lung carcinoma (NSCLC) [45]. In both of our tested cell lines, the levels of p-RPS6 were significantly reduced, which might indicate the importance of this protein in the Rg3-induced effects observed in both cell lines and the potential of involvement of IGF-1R and glucose metabolism. The role of Rg3 with IGF-1 was previously studied and it was shown that Rg3 decreased the expression of IGF-1, causing a reduced cell proliferation in multiple myeloma and breast tumours (reviewed in [9]). This could further highlight the role Rg3 in IGF-1R signalling.

p-PRAS40 is an important component and regulator of mTORC1. The expression of p-PRAS40 has been reported to be prognostic in gastric cancer [46] and prostate cancer [47] patients. Raf-1 and RSK1 are activated downstream of activation of the Ras/Raf/MEK/ERK pathway. RSKs, via enhancing proliferation, migration and invasion, have multiple roles in breast cancer [48]. RSK inhibitors were developed to bypass the side effects of MEK inhibitors, as RSKs have fewer but important targets which play roles in metastasis. The fact that 85% of these patients have activated RSKs makes them a good therapeutic target in TNBC [49].

In addition, the leading edge of migrating cells has a crucial role in cell migration and is a centre for local translation of required proteins for cell migration [50], for which mTOR plays a central role [51]. AQP1, together with other proteins important in cell migration such as focal adhesion kinase (FAK), work in complex at the leading edge to facilitate cell migration. Our preliminary testing has shown that C3 caused decreased activation of FAK and minor decreased expression of AQP1 in MDA-MB-231 and HCC1143 mammospheres (Supplementary Figure S6). A decreased level of AQP1 expression was previously noted in endothelial cells exposed to C3 [15].

The efficacy of C3 was also evaluated in an mTNBC mouse model. Previously some studies reported the efficacy of Rg3 in mice breast cancer models (reviewed in [9]). In these studies, orally administered Rg3 promoted the anti-cancer effects of paclitaxel and capecitabin, and improved mice survival (reviewed in [9]). Although many studies suggested anti-migration mechanisms for Rg3 epimers and anti-metastatic efficacy of Rg3 was shown in melanoma and colon cancer models (reviewed in [9]), until now, no studies have investigated the efficacy of Rg3 on an mTNBC mouse model. For the first time, we have evaluated the efficacy of Rg3 combination in a highly aggressive orthotopic mouse model of mTNBC. Furthermore, we changed the route of administration from oral to subcutaneous injection, hence bypassing the gastrointestinal metabolism, first pass effect and low bioavailability of Rg3 (reviewed in [9]). The primary and the increased dosing both showed efficacy in this mouse model. Reduction in tumour size was more meaningful with the escalated dosing, although the percentage of necrotic tissue between the two groups was not significantly different. Mollard et al. (2016), in a simulation study, showed that in this mTNBC mouse model, necrotic tissue had an almost constant ratio against total tissue volume [52]. Consistent with that, combo B did not change the ratio between necrotic and proliferative tissues in vehicle or treated groups, but primary tumour shrinkage was observed. This could mean that the treatment reduced the rate of proliferation in the primary tumour, which caused tumour shrinkage. Previously, we showed that at 100 µM SRg3, proliferation of MDA-MB-231 was inhibited [11]. Therefore, equivalent dose of 100 µM SRg3 in combo B could play a role in inhibition of proliferation of this tumour. We also have shown that C3 has antiangiogenic properties in vitro [15]. Further studies are recommended to investigate the in vivo antiangiogenic properties of this combination.

In addition, it has been shown that CD44^+^/CD24^−^ subpopulation of breast cancer cells enhance the lung metastasis capacity due to their stem cell-like, progenitor and highly invasive characteristics [53]. We showed that C3 reduces the expression of this phenotype in vitro. Further experiments are suggested to investigate the expression of CD44^+^/CD24^−^ subpopulation in the lung metastatic nodules. Furthermore, epimers of ginsenoside Rh2 and protopanaxadiol were suggested as potential active metabolites of Rg3, detected after intravenous injection to rats (reviewed in [9]), and these molecules have shown anticancer effects in MDA-MB-231 [54,55]. For example, the anti-cancer effects of protopanaxadiol were shown in a less aggressive mouse model of TNBC [56]. Therefore, the effects observed in this study might be a result of combination of Rg3 and its metabolites, which could improve the outcome by introducing several anti-cancer agents to the tumour. Further studies are required to investigate it. For the first time, we reported the anti-metastatic efficacy of combination Rg3 epimers in an mTNBC mouse model. Reduced metastatic load observed in this study is very impressive. Whether this treatment targets angiogenesis in vivo requires further investigation. Furthermore, the treatment was tolerable in mice and no signs of toxicity was observed. Single epimers administered to human have not shown toxicities (reviewed in [9]) and therefore, it is not expected that the tested combination be any more toxic.

## 4. Materials and Methods

### 4.1. Materials

SRg3 and RRg3 (both with a purity of >98% by HPLC) were purchased from ChemFaces^®®^, Wuhan, China. Stocks of SRg3 and RRg3 were prepared in dimethyl sulfoxide (DMSO D2650, HYBRI-MAX, Sigma-Aldrich, Steinheim, Germany) and stored at −20 °C, as previously described [11]. TNBC cell lines, MDA-MB-231 (basal-like with mesenchymal or claudin-low phenotype) and HCC1143 (basal-like with epithelial phenotype) and MCF-12A (immortalised normal human breast epithelial cell line) were purchased from American Type Culture Collection (ATCC), (Manassas, VA, USA). Luciferase-expressing MDA-MB-231-Luc cells were purchased from CellBank Australia (Westmead, NSW). All cells were mycoplasma-free. As previously described, cells were tested for mycoplasma using MycoAlert Detection Kit (Lonza, Basel, Switzerland) and/or a custom PCR-based assay [57,58].

### 4.2. Cell Culture of Adherent Cells

MDA-MB-231 cells were grown in Dulbecco’s Modified Eagle Medium (DMEM; Life Technologies, CA, USA) and HCC1143 cells were grown in Roswell Park Memorial Institute (RPMI) 1640 medium (Life Technologies, Carlsbad, CA, USA). Media was supplemented with 10% foetal bovine serum (FBS; Corning, Corning, NY, USA) and 1% penicillin-streptomycin solution (Life Technologies). MCF-12A cells were grown in MammoCult™ media supplemented with 5% FBS (Corning, NY, USA), 10% MammoCult™ proliferation supplement, 0.5% hydrocortisone and 0.2% heparin (all from Stem Cell Technologies, Vancouver, BC, Canada). MDA-MB-231-Luc cells were cultured in L-15 media supplemented with 15% FBS and 1% penicillin-streptomycin (Life Technologies, Carlsbad, CA, USA). A week before the animal experiment, the cells were harvested, washed in Dulbecco’s phosphate-buffered saline (DPBS, Gibco, Thermo Fisher Scientific, Waltham, MA, USA) and grown as mammospheres in MammoCult™ media (described below).

### 4.3. Circular Scratch Migration Assay

The experiment was performed as previously described [11]. Briefly, three-day pre-treated MDA-MB-231 and HCC-1143 cells were seeded in 96-well cell culture plates (Corning^®®^ Costar^®®^, Corning, NY, USA), at 4 × 10^4^ and 8 × 10^4^ cells/well, respectively. Following overnight incubation, a circular scratch was made on a monolayer of cells and images at time 0 and 24 h were taken using a Nikon microscope. The migration (%) of the cells was measured based on the area of the circular wound measured with ImageJ software (version 1.53a, National Health of Institute, Bethesda, MD, USA) for each well as a percentage of the initial wound area at time 0. The experiment was replicated 6 times and results represented as mean ± standard deviation (SD).

### 4.4. Response Surface Methodology (RSM)

To confirm that the effective combination of Rg3 epimers on endothelial cells was effective on TNBC cells, an RSM model was developed for the migration of MDA-MB-231, as previously described (Supplementary Tables S2 and S3) [15]. The tested combinations were 12.5 µM SRg3 and 6.2 µM RRg3 (C1), 25 µM SRg3 and 12.5 µM RRg3 (C2), and 50 µM SRg3 and 25 µM RRg3 (C3).

### 4.5. Transwell Migration Assay

The experiment was performed as previously described [11]. A total of 1 × 10^5^ of pre-treated MDA-MB-231 and HCC-1143 cells were collected in 250 μL of serum-free media containing vehicle control or Rg3 combinations. The cells were added to the upper chamber of Corning^®®^ transwell plates (8 µm pore size) with complete media in the lower chamber. The experiment was performed in triplicate and the results were represented as mean ± SD.

### 4.6. Proliferation Assay

Crystal violet assay (CVA) was used to study the effect of Rg3 on the proliferation of TNBC cells, as previously described [11]. Briefly, MDA-MB-231, HCC1143 and MCF-12A cells were seeded at 5 × 10^3^ cells/well of 96-well flat-bottom cell culture plates. After overnight incubation, the cells were exposed to the vehicle or Rg3 combinations. On days 0 (drug exposure day), 1 and 3, cells were stained with crystal violet and the absorbance was measured at 595 nm using FLUOstar Optima microplate reader (BMG Labtech, Offenburg, Germany). Each treatment replicated 6 times and the data were shown as mean ± SD.

### 4.7. Culture of Mammospheres

Mammospheres were grown in MammoCult™ Human Medium Kit (Stem Cell Technologies, Vancouver, Canada) as recommended by the manufacturer. The media was supplemented with MammoCult™ proliferation supplement, hydrocortisone and heparin at final concentrations of 10%, 0.5% and 0.2%, respectively. Briefly, the cells were harvested, washed and resuspended in complete MammoCult media at the density of 4 × 10^3^ cell/cm^2^ in 6-well ultra-low attachment plates (Corning^®®^ Costar^®®^, Corning, NY, USA). Following a 7-day period, mammospheres were collected in a tube, centrifuged at 350× *g*, 10 min, 4 °C, and dissociated using 100 µL TrypLE™ Express (phenol red-free, Gibco, Thermo Fisher Scientific, Waltham, MA, USA) and pipetting. Then, 1 mL of cold DPBS supplemented with 2% FBS was added to the cells. The cells were then centrifuged at 350× *g*, 10 min, 4 °C. Supernatant was then removed, and single cells were suspended in MammoCult media, counted and seeded in 6-well ultra-low attachment plates exposed with vehicle or C3 for 3 days.

### 4.8. Mammosphere Formation Efficiency (MFE)

Mammospheres, exposed to vehicle or Rg3, were collected, counted and seeded in 24-well ultra-low attachment plates at the density of 4000 cell/cm^2^. They were exposed with the vehicle or Rg3 for 4 days. Then, MFE was calculated as per the equation MFE (%) = number of mammospheres per well/number of seeded cells per well ×100, as previously described [59].

### 4.9. Cell Viability Analysis

Cells were grown and exposed with the drugs as described in the previous paragraph. Single cells were prepared, and cell viability was measured using CellDrop™ FL Fluorescence Cell Counter (DeNOVIX). The experiment was performed in triplicate and the results are expressed as mean ± SD.

### 4.10. Expression of Stem Cell Markers

Mammospheres were grown, treated with C3, collected and dissociated into single cells as before. Then, the cells were washed in cold fluorescence-activated cell sorting buffer (FACS, 2% FBS, 0.05% sodium azide in DPBS), counted and 1 × 10^6^ cell/mL FACS was prepared. The antibodies used to study the expression of CD44 and CD24 markers were as follows: APC mouse anti-human CD44 (BD Pharmingen™, BD Biosciences, CA, USA), APC mouse IgG2b, κ isotype control (BD Pharmingen™, BD Biosciences, CA, USA), PE mouse anti-human CD24 (BD Pharmingen™, BD Biosciences, CA, USA) and PE mouse IGg2a, κ isotype control (BD Pharmingen™, BD Biosciences, CA, USA). APC-Cy7 (100 µL per tube) was used to select for live cells. Incubation with antibodies or isotype controls was for 30 min in darkness at 4 °C. Cells were washed twice in 1 mL FACS buffer and resuspended in 100 µL of FACS buffer. Expression of ALDH was studied using ALDEFLUOR™ Kit (Stem Cell Technologies), based on the manufacturer’s protocol. An aliquot of 1 × 10^5^ cells/tube was prepared on ice for 30 min staining with 20 µL antibody or isotype control. Prior to analysis, the cells were washed twice in 1 mL FACS buffer and resuspended in 100 µL of FACS buffer. The experiments were performed in triplicate and the results are expressed as mean ± SD.

### 4.11. AKT Pathway Phosphorylation Array

A Human/Mouse AKT Pathway Phosphorylation Array C1 (RayBiotech, Norcross, GA, USA) was used to evaluate the effect of C3 on the signalling of AKT. This array measures the expression of 18 phosphorylated proteins in this signalling pathway. Mammospheres were grown from both TNBC cell lines, exposed to drugs and collected as described above. Mammospheres were washed twice in cold DPBS. Protein was extracted with a protein inhibitor cocktail and phosphatase inhibitors included as recommended by the manufacturer. Bio-Rad Protein Assay Dye Reagent Concentrate (Bio-Rad, Hercules, CA, USA) was used for the determination of protein levels. Image Lab™ Software (version 6.1) was used to measure the density of each dot. The results are shown as the mean ± SD of two replicates.

### 4.12. In Silico Molecular Docking

The interaction of Rg3 with four tyrosine kinase receptors was studied via molecular docking. The SMILES structure of Rg3 isomers, sorafenib, lenvatinib and rapamycin were obtained from PubChem. The crystal structure of EGFR (3W33), HER-2 (3PP0), IGF-1R (1JQH), PDGFR (5GRN), FRB (1FAP) and Rheb (6BSX) were obtained from the protein data bank of NCBI (RCSB PDB). The UCSF Chimera program (version 1.15-mac64) and Autodock Vina algorithm (version 1.1.2_Mac) were used to create the 3D structure of Rg3 and perform the molecular docking. The Gibbs free energy of protein–ligand binding (kJ/mol) was predicted based on the flexible ligand docking simulations within the docking grids on defined interaction sites of each specific protein, as previously described [11].

### 4.13. Developing the Mouse Model of mTNBC

Female Nod *scid* gamma (NSG) mice (6–8 weeks old) were purchased from Animal Resources Centre (Canning Vale, WA, Australia). Five days prior to commencement of the study, the mice were acclimatized to the animal housing facility, in pathogen-free conditions. Throughout the study, the mice were weighed and checked on a daily basis for general well-being or any signs of toxicity. The experiments were performed according to the guidelines for ethical use of animals in research and were approved by the Animal Ethics Committees of the University of Adelaide (M-2019-068, 01/08/2019). To prepare MDA-MB-231-Luc cells for injection into mice, they were grown as mammospheres for 7 days. On the day of cell inoculation, the cells were collected, centrifuged at 350× *g* for 5 min at 4 °C and washed twice in DPBS. The cells were counted and 1 × 10^6^ cells (viability > 95%) were resuspended in 50 µL cold DPBS. A total of 100 µL of cells in a 1:1 ratio of Matrigel (Corning^®®^ Matrigel^®®^ Basement Membrane Matrix, Phenol red-free, LDEV-free)/DPBS were injected into the 4th right MFP, using aseptic technique.

### 4.14. Drug Administration and Toxicity Assessment

Drugs were dissolved in DMSO and stored in −20 °C as aliquots. Before injection to mice, an aliquot was thawed, mixed with 70% DPBS and 20% Cremophor EL^®®^ (Millipore Corp., Billerica, MA, USA). The vehicle group received a combination of 10% DMSO, 20% Cremophor EL and 70% DPBS. Doses administered to mice were an extrapolation of in vitro results considering the total body water volume (12.65 mL) in a mouse of an average weight of 22 g. In the first study (study A), the mice received a combination of 50 µM SRg3 and 25 µM RRg3, equivalent to 23 and 11 mg/kg (combo A), respectively. Based on the encouraging results of this study, study B was designed in which the mice received a combination of 100 µM SRg3 and 50 µM RRg3, equivalent to 45 and 23 mg/kg (combo B), respectively. Drug administration started on day 12 in study A and day 5 in study B. Studies A and B lasted for 40 and 23 days, respectively. The drugs were injected subcutaneously three times a week. To assess the toxicity of the treatment, the mice were checked daily for any adverse changes including changes in body weight, changes in behaviour, fur condition, movement, posture, breathing and hydration level.

### 4.15. IVIS Imaging and Tumour Size Measurement

To non-invasively monitor the growth of the tumour and development of metastasis, the IVIS^®®^ Spectrum Imaging system (PerkinElmer, Boston, MA, USA) was used. The mice were anaesthetised and given a subcutaneous injection of 100 µL of luciferin solution (Xenolight™ D-luciferin potassium salt, PerkinElmer) at 150 mg/kg, 20 min before imaging. Mice were imaged once a week. Living Image^®®^ software (version 4.7.3; Perkin Elmer) was used to quantify the photons emitted from the mice as photons/sec/cm^2^. Furthermore, calliper measurement was used to measure the volume of the primary tumour using the equation: (shortest diameter of the tumour)^2^ × longest diameter of the tumour/2. Rate of tumour growth was calculated as follows:

$$\mathrm{Rate}\;\mathrm{of}\;\mathrm{tumour}\;\mathrm{growth}\%=\frac{Total\;flux\;on\;day_{n}-First\;measured\;total\;flux}{First\;measured\;total\;flux}\times 100$$

### 4.16. H&E Staining and Proliferative Area Measurement

At the end of the experiment, the mice were humanely euthanised. Primary tumours and organs including lungs and lymph nodes were fixed in formalin neutral buffered, 10%, histological tissue fixative (Sigma-Aldrich) for 2 days, dehydrated and wax infiltrated (Excelsior AS™ Tissue Processor, Thermo Scientific™) and embedded in paraffin (HistoStar™ Embedding Workstation, Thermo Scientific™). Then, 4 µm thick tissue sections were prepared using a Microm HM 325 Microtome. Tissue slides were then stained for H&E, imaged using a NanoZoomer 2.0-HT slide scanner and viewed with NDP.view2 software (Hamamatsu Photonics, Shizuoka, Japan). The area of necrotic tissue relative to the whole cross-sectional area of the tumours was measured using ImageJ.

### 4.17. Statistical Analysis

GraphPad Prism (version 9.0.0 for Mac, GraphPad Software, San Diego, CA, USA) was used to perform Student’s *t*-test, one- or two-way analysis of variance (repeated measurement) or Chi-squared test. The significance cut-off was considered to be *p* < 0.05.

## 5. Conclusions

In conclusion, the optimised combination of SRg3 + RRg3 showed efficacy in inhibition of migration of TNBC cell lines, inhibited the MFE, reduced the ‘stemness’ of these cells and inhibited phosphorylation of proteins downstream of AKT/mTOR signalling. In silico studies suggest that Rg3 might be a potential mTORC1 inhibitor. Furthermore, in an mTNBC mouse model, the treatment shrunk the primary tumour and reduced the load of metastasis in mice, which introduces this drug as a potential treatment for TNBC.

## Figures and Tables

**Table 1:** Binding score (kJ/mol) between Rg3 epimers and tyrosine kinase receptors, the FKBP12 rapamycin-binding (FRB) site of mTOR or Ras homolog enriched in brain (Rheb), and the number of hydrogen bonds (H-bonds) predicted by Chimera program and Autodock vina algorithm.

	Binding Score (kJ/mol) (Number of H-Bonds)					
Molecule	EGFR	HER-2	IGF-1R	PDGFR	FRB	Rheb
SRg3	−6.9 (2)	2.7 (0)	−8.0 (2)	−2.8 (1)	−7.0 (1)	−8.0 (3)
RRg3	−6.9 (2)	2.7 (1)	−7.5 (0)	−2.8 (1)	−7.2 (1)	−8.0 (4)
Sorafenib	−9.6 (0)	−10.8 (1)	−8.9 (1)	−11.2 (1)	n.d. ^1^	n.d.
Lenvatinib	−10.4 (1)	−9.6 (1)	−7.9 (1)	−10.1 (1)	n.d.	n.d.
Rapamycin	n.d.	n.d.	n.d.	n.d.	−7.6 (0)	n.d.

^1^ not determined.

## Data Availability

All data are available in the paper or as supplementary data.

## Supplementary Materials

The following are available online at https://www.mdpi.com/article/10.3390/ph14070633/s1.

## References

[1] Garrido-Castro A.C., Lin N.U., Polyak K. (2019). Insights into molecular classifications of triple-negative breast cancer: Improving patient selection for treatment. Cancer Discov..

[2] Biswas T., Efird J.T., Prasad S., Jindal C., Walker P.R. (2017). The survival benefit of neoadjuvant chemotherapy and pCR among patients with advanced stage triple negative breast cancer. Oncotarget.

[3] Groza I.-M., Braicu C., Jurj A., Zanoaga O., Lajos R., Chiroi P., Cojocneanu R., Paun D., Irimie A., Korban S.S. (2020). Cancer-Associated Stemness and Epithelial-to-Mesenchymal Transition Signatures Related to Breast Invasive Carcinoma Prognostic. Cancers.

[4] Li W., Ma H., Zhang J., Zhu L., Wang C., Yang Y. (2017). Unraveling the roles of CD44/CD24 and ALDH1 as cancer stem cell markers in tumorigenesis and metastasis. Sci. Rep..

[5] Nakhjavani M., Hardingham J.E., Palethorpe H.M., Price T.J., Townsend A.R. (2019). Druggable Molecular Targets for the Treatment of Triple Negative Breast Cancer. J. Breast Cancer.

[6] Xia P., Xu X.-Y. (2015). PI3K/Akt/mTOR signaling pathway in cancer stem cells: From basic research to clinical application. Am. J. Cancer Res..

[7] Li X., Zhou N., Wang J., Liu Z., Wang X., Zhang Q., Liu Q., Gao L., Wang R. (2018). Quercetin suppresses breast cancer stem cells (CD44+/CD24−) by inhibiting the PI3K/Akt/mTOR-signaling pathway. Life Sci..

[8] Guerrero-Zotano A., Mayer I.A., Arteaga C.L. (2016). PI3K/AKT/mTOR: Role in breast cancer progression, drug resistance, and treatment. Cancer Metastasis Rev..

[9] Nakhjavani M., Hardingham J.E., Palethorpe H.M., Tomita Y., Smith E., Price T.J., Townsend A.R. (2019). Ginsenoside Rg3: Potential molecular targets and therapeutic indication in metastatic breast cancer. Medicines.

[10] Nakhjavani M., Smith E., Townsend A.R., Price T.J., Hardingham J.E. (2020). Anti-Angiogenic Properties of Ginsenoside Rg3. Molecules.

[11] Nakhjavani M., Palethorpe H.M., Tomita Y., Smith E., Price T.J., Yool A.J., Pei J.V., Townsend A.R., Hardingham J.E. (2019). Stereoselective anti-cancer activities of ginsenoside Rg3 on triple negative breast cancer cell models. Pharmaceuticals.

[12] Aboushady D., Parr M.K., Hanafi R.S. (2020). Quality-by-Design Is a Tool for Quality Assurance in the Assessment of Enantioseparation of a Model Active Pharmaceutical Ingredient. Pharmaceuticals.

[13] Zhao W., Sachsenmeier K., Zhang L., Sult E., Hollingsworth R.E., Yang H. (2014). A new bliss independence model to analyze drug combination data. J. Biomol. Screen..

[14] Chui C.H., Wong R.S.M., Cheng G.Y.M., Lau F.Y., Kok S.H.L., Cheng C.H., Cheung F., Tang W.K., Teo I.T.N., Chan A.S.C. (2006). Antiproliferative ability of a combination regimen of crocodile egg extract, wild radix ginseng and natural Ganoderma lucidum on acute myelogenous leukemia. Oncol. Rep..

[15] Nakhjavani M., Smith E., Yeo K., Palethorpe H.M., Tomita Y., Price T.J., Townsend A.R., Hardingham J.E. (2021). Anti-Angiogenic Properties of Ginsenoside Rg3 Epimers: In Vitro Assessment of Single and Combination Treatments. Cancers.

[16] Grimshaw M.J., Cooper L., Papazisis K., Coleman J.A., Bohnenkamp H.R., Chiapero-Stanke L., Taylor-Papadimitriou J., Burchell J.M. (2008). Mammosphere culture of metastatic breast cancer cells enriches for tumorigenic breast cancer cells. Breast Cancer Res..

[17] Yousefnia S., Ghaedi K., Seyed Forootan F., Nasr Esfahani M.H. (2019). Characterization of the stemness potency of mammospheres isolated from the breast cancer cell lines. Tumor Biol..

[18] Zou W., Yang Y., Zheng R., Wang Z., Zeng H., Chen Z., Yang F., Wang J. (2020). Association of CD44 and CD24 phenotype with lymph node metastasis and survival in triple-negative breast cancer. Int. J. Clin. Exp. Pathol..

[19] Hiraga T., Ito S., Nakamura H. (2011). Side population in MDA-MB-231 human breast cancer cells exhibits cancer stem cell-like properties without higher bone-metastatic potential. Oncol. Rep..

[20] Masuda H., Zhang D., Bartholomeusz C., Doihara H., Hortobagyi G.N., Ueno N.T. (2012). Role of epidermal growth factor receptor in breast cancer. Breast Cancer Res. Treat..

[21] Bahhnassy A., Mohanad M., Shaarawy S., Ismail M.F., El-Bastawisy A., Ashmawy A.M., Zekri A.R. (2015). Transforming growth factor-β, insulin-like growth factor I/insulin-like growth factor I receptor and vascular endothelial growth factor-A: Prognostic and predictive markers in triple-negative and non-triple-negative breast cancer. Mol. Med. Rep..

[22] Toyama T., Yamashita H., Kondo N., Okuda K., Takahashi S., Sasaki H., Sugiura H., Iwase H., Fujii Y. (2008). Frequently increased epidermal growth factor receptor (EGFR) copy numbers and decreased BRCA1 mRNA expression in Japanese triple-negative breast cancers. BMC Cancer.

[23] Wu C.-W., Storey K.B. (2012). Regulation of the mTOR signaling network in hibernating thirteen-lined ground squirrels. J. Exp. Biol..

[24] Ruvinsky I., Sharon N., Lerer T., Cohen H., Stolovich-Rain M., Nir T., Dor Y., Zisman P., Meyuhas O. (2005). Ribosomal protein S6 phosphorylation is a determinant of cell size and glucose homeostasis. Genes Dev..

[25] Romeo Y., Zhang X., Roux P.P. (2012). Regulation and function of the RSK family of protein kinases. Biochem. J..

[26] Anjum R., Blenis J. (2008). The RSK family of kinases: Emerging roles in cellular signalling. Nat. Rev. Mol. Cell Biol..

[27] Hekman M., Fischer A., Wennogle L.P., Wang Y.K., Campbell S.L., Rapp U.R. (2005). Novel C-Raf phosphorylation sites: Serine 296 and 301 participate in Raf regulation. FEBS Lett..

[28] Cardillo T.M., Trisal P., Arrojo R., Goldenberg D.M., Chang C.-H. (2013). Targeting both IGF-1R and mTOR synergistically inhibits growth of renal cell carcinoma in vitro. BMC Cancer.

[29] Lamhamedi-Cherradi S.-E., Menegaz B.A., Ramamoorthy V., Vishwamitra D., Wang Y., Maywald R.L., Buford A.S., Fokt I., Skora S., Wang J. (2016). IGF-1R and mTOR blockade: Novel resistance mechanisms and synergistic drug combinations for Ewing sarcoma. J. Natl. Cancer Inst..

[30] Ji P., Zhang Y., Wang S.-J., Ge H.-L., Zhao G.-P., Xu Y.-C., Wang Y. (2016). CD44hiCD24lo mammosphere-forming cells from primary breast cancer display resistance to multiple chemotherapeutic drugs. Oncol. Rep..

[31] Giatromanolaki A., Sivridis E., Fiska A., Koukourakis M.I. (2011). The CD44+/CD24− phenotype relates to ‘triple-negative’state and unfavorable prognosis in breast cancer patients. Med. Oncol..

[32] Shipitsin M., Campbell L.L., Argani P., Weremowicz S., Bloushtain-Qimron N., Yao J., Nikolskaya T., Serebryiskaya T., Beroukhim R., Hu M. (2007). Molecular definition of breast tumor heterogeneity. Cancer Cell..

[33] Abraham B.K., Fritz P., McClellan M., Hauptvogel P., Athelogou M., Brauch H. (2005). Prevalence of CD44+/CD24−/low cells in breast cancer may not be associated with clinical outcome but may favor distant metastasis. Clin. Cancer Res..

[34] Sheridan C., Kishimoto H., Fuchs R.K., Mehrotra S., Bhat-Nakshatri P., Turner C.H., Goulet R., Badve S., Nakshatri H. (2006). CD44+/CD24-breast cancer cells exhibit enhanced invasive properties: An early step necessary for metastasis. Breast Cancer Res..

[35] Van Phuc P., Nhan P.L.C., Nhung T.H., Tam N.T., Hoang N.M., Tue V.G., Thuy D.T., Ngoc P.K. (2011). Downregulation of CD44 reduces doxorubicin resistance of CD44+ CD24− breast cancer cells. OncoTargets Ther..

[36] Oh J., Yoon H.-J., Jang J.-H., Kim D.-H., Surh Y.-J. (2019). The standardized Korean Red Ginseng extract and its ingredient ginsenoside Rg3 inhibit manifestation of breast cancer stem cell–like properties through modulation of self-renewal signaling. J. Ginseng Res..

[37] Xu H., Tian Y., Yuan X., Wu H., Liu Q., Pestell R.G., Wu K. (2015). The role of CD44 in epithelial–mesenchymal transition and cancer development. Onco Targets Ther..

[38] Chen J., Shao R., Li F., Monteiro M., Liu J.P., Xu Z.P., Gu W. (2015). PI 3K/Akt/mTOR pathway dual inhibitor BEZ 235 suppresses the stemness of colon cancer stem cells. Clin. Exp. Pharmacol. Physiol..

[39] Lu C., Makala L., Wu D., Cai Y. (2016). Targeting translation: eIF4E as an emerging anticancer drug target. Expert Rev. Mol. Med..

[40] Rutkovsky A.C., Yeh E.S., Guest S.T., Findlay V.J., Muise-Helmericks R.C., Armeson K., Ethier S.P. (2019). Eukaryotic initiation factor 4E-binding protein as an oncogene in breast cancer. BMC Cancer.

[41] Pons B., Peg V., Vázquez-Sánchez M.Á., López-Vicente L., Argelaguet E., Coch L., Martínez A., Hernández-Losa J., Armengol G., Ramon y Cajal S. (2011). The effect of p-4E-BP1 and p-eIF4E on cell proliferation in a breast cancer model. Int. J. Oncol..

[42] Hartman N.W., Lin T.V., Zhang L., Paquelet G.E., Feliciano D.M., Bordey A. (2013). mTORC1 Targets the Translational Repressor 4E-BP2, but Not S6 Kinase 1/2, to Regulate Neural Stem Cell Self-Renewal In Vivo. Cell Rep..

[43] Guo L., Abraham J., Flynn D., Castranova V., Shi X., Qian Y. (2007). Individualized survival and treatment response predictions for breast cancers using phospho-EGFR, phospho-ER, phospho-HER2/neu, phospho-IGF-IR/In, phospho-MAPK, and phospho-p70S6K proteins. Int. J. Biol. Marker..

[44] Xiao L., Wang Y.C., Li W.S., Du Y. (2009). The role of mTOR and phospho-p70S6K in pathogenesis and progression of gastric carcinomas: An immunohistochemical study on tissue microarray. J. Exp. Clin. Cancer Res..

[45] Chen B., Tan Z., Gao J., Wu W., Liu L., Jin W., Cao Y., Zhao S., Zhang W., Qiu Z. (2015). Hyperphosphorylation of ribosomal protein S6 predicts unfavorable clinical survival in non-small cell lung cancer. J. Exp. Clin. Cancer Res..

[46] Lu Y.-Z., Deng A.-M., Li L.-H., Liu G.-Y., Wu G.-Y. (2014). Prognostic role of phospho-PRAS40 (Thr246) expression in gastric cancer. Arch. Med. Res..

[47] Shipitsin M., Small C., Giladi E., Siddiqui S., Choudhury S., Hussain S., Huang Y.E., Chang H., Rimm D.L., Berman D.M. (2014). Automated quantitative multiplex immunofluorescence in situ imaging identifies phospho-S6 and phospho-PRAS40 as predictive protein biomarkers for prostate cancer lethality. Proteome Sci..

[48] Zhao H., Martin T.A., Davies E.L., Ruge F., Yu H., Zhang Y., Teng X., Jiang W.G. (2016). The Clinical Implications of RSK1-3 in Human Breast Cancer. Anticancer Res..

[49] Ludwik K.A., Campbell J.P., Li M., Li Y., Sandusky Z.M., Pasic L., Sowder M.E., Brenin D.R., Pietenpol J.A., O’Doherty G.A. (2016). Development of a RSK inhibitor as a novel therapy for triple-negative breast cancer. Mol. Cancer Ther..

[50] Herbert S.P., Costa G. (2019). Sending messages in moving cells: mRNA localization and the regulation of cell migration. Essays. Biochem..

[51] Willett M., Brocard M., Davide A., Morley S.J. (2011). Translation initiation factors and active sites of protein synthesis co-localize at the leading edge of migrating fibroblasts. Biochem. J..

[52] Mollard S., Fanciullino R., Giacometti S., Serdjebi C., Benzekry S., Ciccolini J. (2016). In vivo bioluminescence tomography for monitoring breast tumor growth and metastatic spreading: Comparative study and mathematical modeling. Sci. Rep..

[53] Hu J., Li G., Zhang P., Zhuang X., Hu G. (2017). A CD44v+ subpopulation of breast cancer stem-like cells with enhanced lung metastasis capacity. Cell Death Dis..

[54] Choi S., Kim T.W., Singh S.V. (2009). Ginsenoside Rh2-mediated G 1 phase cell cycle arrest in human breast cancer cells is caused by p15 Ink4B and p27 Kip1-dependent inhibition of cyclin-dependent kinases. Pharm. Res..

[55] Kwak J.H., Park J.Y., Lee D., Kwak J.Y., Park E.H., Kim K.H., Park H.-J., Kim H.Y., Jang H.J., Ham J. (2014). Inhibitory effects of ginseng sapogenins on the proliferation of triple negative breast cancer MDA-MB-231 cells. Bioorganic Med. Chem. Lett..

[56] Peng B., He R., Xu Q., Yang Y., Hu Q., Hou H., Liu X., Li J. (2019). Ginsenoside 20(S)-protopanaxadiol inhibits triple-negative breast cancer metastasis in vivo by targeting EGFR-mediated MAPK pathway. Pharmacol. Res..

[57] Smith E., Palethorpe H.M., Tomita Y., Pei J.V., Townsend A.R., Price T.J., Young J.P., Yool A.J., Hardingham J.E. (2018). The Purified Extract from the Medicinal Plant Bacopa monnieri, Bacopaside II, Inhibits Growth of Colon Cancer Cells In Vitro by Inducing Cell Cycle Arrest and Apoptosis. Cells.

[58] Paltoglou S., Das R., Townley S.L., Hickey T.E., Tarulli G.A., Coutinho I., Fernandes R., Hanson A.R., Denis I., Carroll J.S. (2017). Novel Androgen Receptor Coregulator GRHL2 Exerts Both Oncogenic and Antimetastatic Functions in Prostate Cancer. Cancer Res..

[59] Lombardo Y., de Giorgio A., Coombes C.R., Stebbing J., Castellano L. (2015). Mammosphere formation assay from human breast cancer tissues and cell lines. J. Vis. Exp..

