# Supplementary material for: Anti-Cancer Effects of an Optimised Combination of Ginsenoside Rg3 Epimers on Triple Negative Breast Cancer Models
Source: Pharmaceuticals (Basel). 2021 Jun 30;14(7):633. doi: 10.3390/ph14070633 (PMC8308773; doi:10.3390/ph14070633)
Supplement: Supplementary file 1 [file pharmaceuticals-14-00633-s001.zip › pharmaceuticals-1193028-supplementary.pdf]

**Supplementary Table S1.** Interaction sites of SRg3 and RRg3 with amino acids within IGF-1R, FRB and Rheb, represented from the best binding score out of 10, 9 and 9 conformational positions, respectively.

| Target Molecule | H Bonds with Amino Acids | |
|---|---|---|
| | **SRg3** | **RRg3** |
| IGF-1R | Leu600, Lys628 | n.a. [1] |
| FRB | Tyr2105.B | Tyr195 |
| Rheb | Gly18.A, Lys19.A, Lys120.A | Tyr34, Ser20 (2 bonds), Thr87 |

[1] not applicable.

**Supplementary Table S2.** Low, mid and high values used for RSM model. The central composite design Table 1. 0 and +1, respectively, for input parameters which are concentrations of SRg3 and RRg3. In the present study, the concentration ranged from (0–100 μM) for SRg3 and (0–50 μM) for RRg3 were selected. Supplementary Table 1 represents the values corresponding to low, mid and high bounds of concentrations for Rg3 epimers.

| Parameter | Index | Concentration (μM) | | |
|---|---|---|---|---|
| | | **Lowest Value (−1)** | **Centre Value (0)** | **Highest Value (+1)** |
| SRg3 | A | 0 | 50 | 100 |
| RRg3 | B | 0 | 25 | 50 |

**Supplementary Table S3.** Design Matrix developed for the RSM analysis and the response (percent of cell migration). To optimise the combination of concentrations, the RSM model has reduced the total experiments to 13 iterations, with cell migration being the ‘main measurable target parameter’.

| Run * | A | B | SRg3 (A) | RRg3 (B) | Response (%) |
|---|---|---|---|---|---|
| 1 | −1 | −1 | 0 | 0 | 79.23 |
| 2 | 0 | 0 | 50 | 25 | 18.40 |
| 3 | 0 | 0 | 50 | 25 | 14.19 |
| 4 | 0 | 0 | 50 | 25 | 15.55 |
| 5 | 1 | 0 | 100 | 25 | 57.24 |
| 6 | 0 | 0 | 50 | 25 | 12.09 |
| 7 | −1 | 1 | 0 | 50 | 64.33 |
| 8 | −1 | 0 | 0 | 25 | 79.23 |
| 9 | 0 | −1 | 50 | 0 | 72.40 |
| 10 | 1 | 1 | 100 | 50 | 53.45 |
| 11 | 0 | 1 | 50 | 50 | 63.92 |
| 12 | 0 | 0 | 50 | 25 | 16.14 |
| 13 | 0 | −1 | 100 | 0 | 75.09 |

*See Table S2 for values corresponding to the bounds −1, 0 and 1.

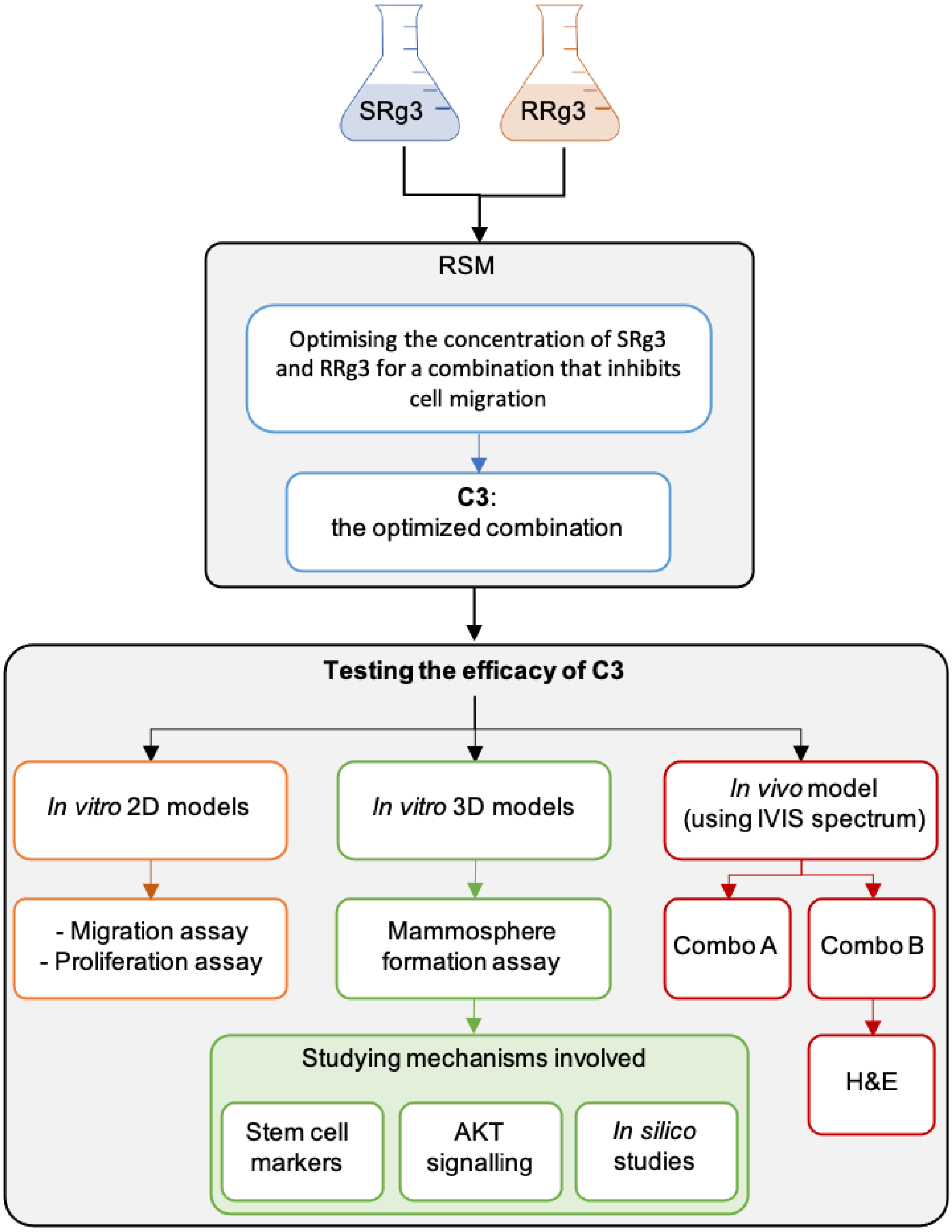


**Supplementary Figure S1.** A diagram depicting the steps undertaken in this study.

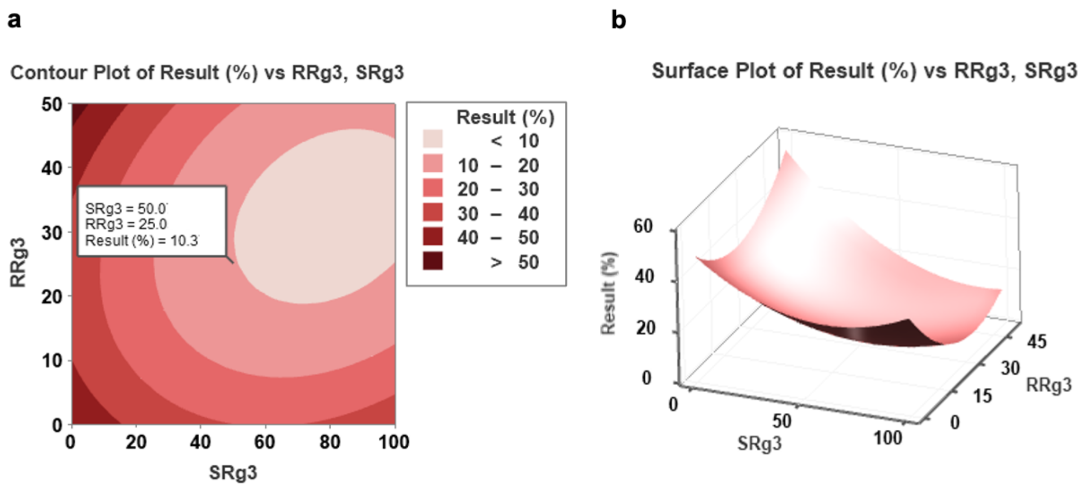


**Supplementary Figure S2.** The calculated (**a**) contour plot and (**b**) 3D surface plots for migration of HCC1143 cell line based on the response surface methodology model developed to show the efficacy of SRg3 + RRg3 drugs in combination.

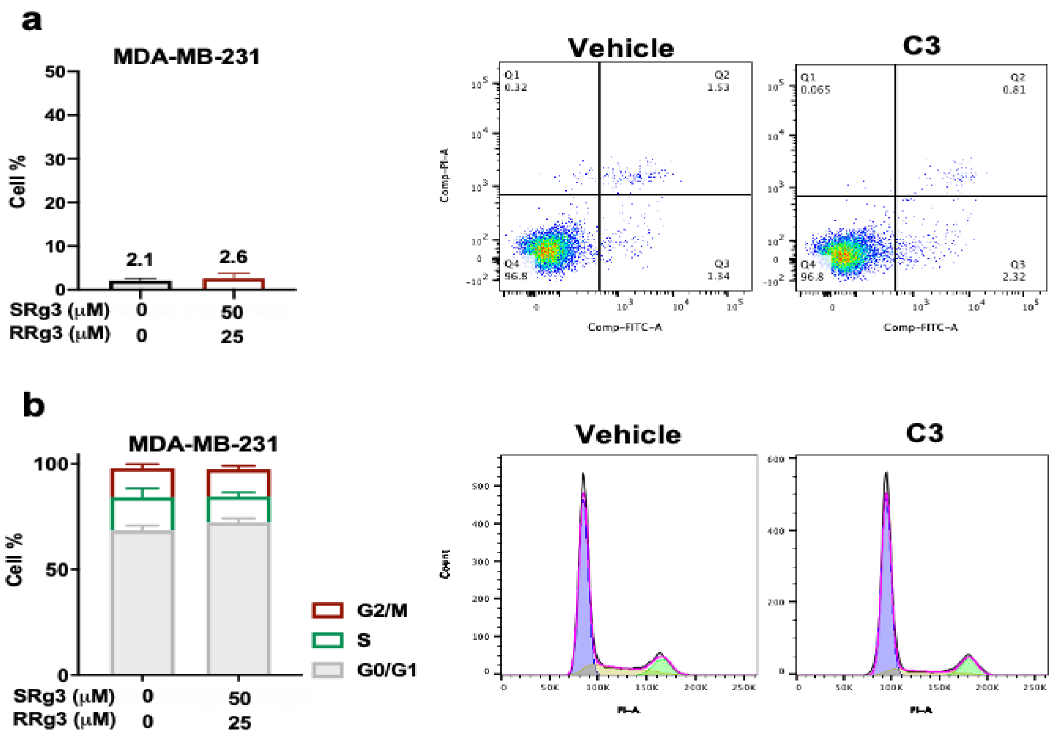


**Supplementary Figure S3.** MDA-MB-231 cells were grown as mammospheres and exposed to C3 (50 μM SRg3 + 25 μM RRg3). (**a**) Apoptotic and (**b**) cell cycle profiles of these mammospheres exposed to vehicle or drug. The experiment was performed in triplicate and results are shown as mean ± SD. Apoptosis assay was performed on mammospheres using Annexin-V-FLUOS staining kit (Roche Diagnostics, Mannheim, Germany) as previously described [1]. Changes in the cell cycle of cells grown as mammospheres were studied using propidium iodide staining and BD FACSCanto™ II analysis, as previously described [2]. The experiment was performed using the BD FACSCanto™ II (BD Biosciences, San Jose, CA, USA) and FlowJo software, v 10.4 (FlowJo, LLC, Ashland, OR, USA).

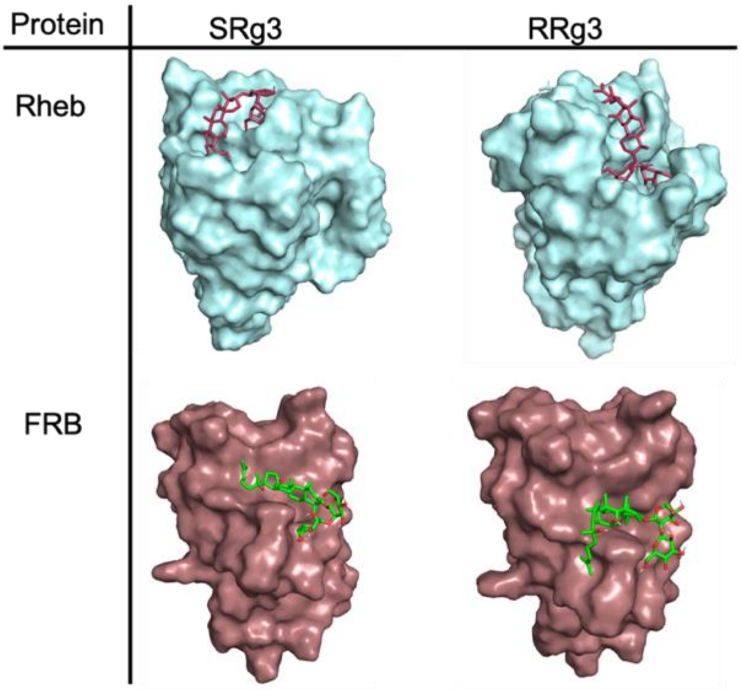


**Supplementary Figure S4.** Binding of SRg3 and RRg3 to Rheb and FRB.

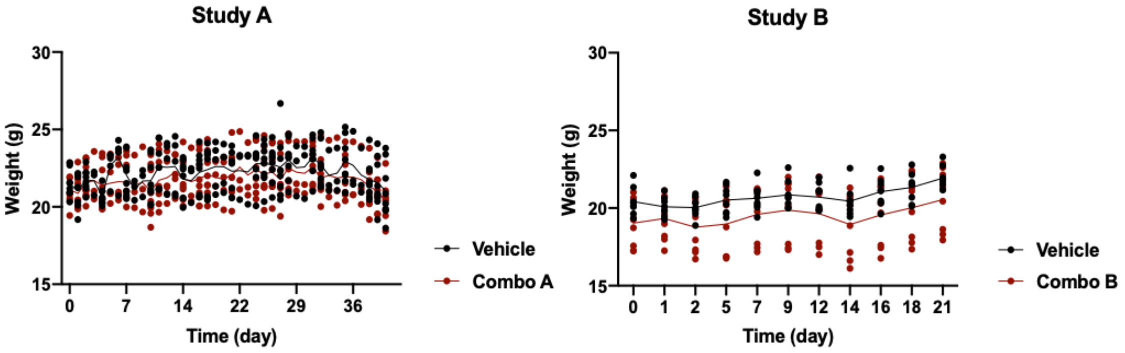


**Supplementary Figure S5.** Weight pattern of mice in the vehicle- or Rg3-treated groups in study A (using combo A) and B (using combo B). There were no significant differences between the groups in terms of changes in body weight.

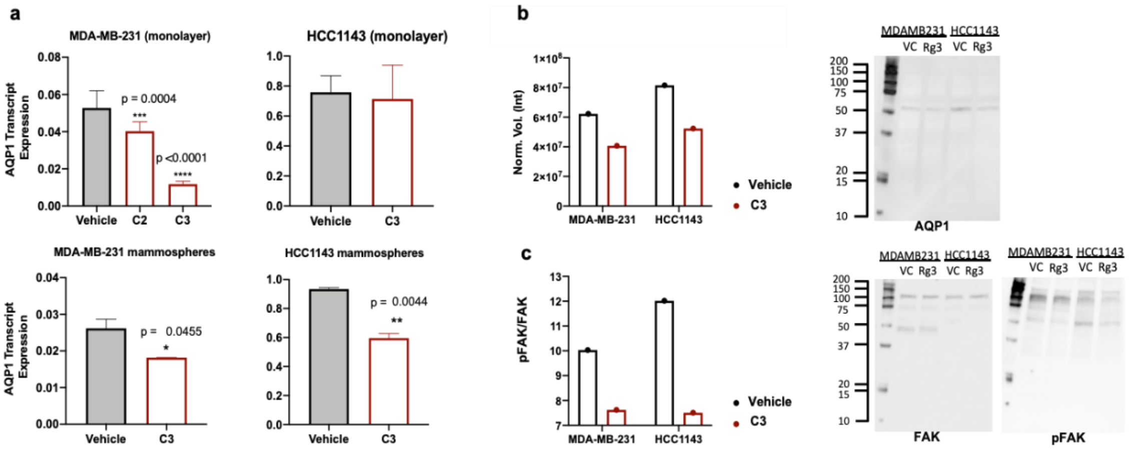


**Supplementary Figure S6.** (**a**) Expression of AQP1 transcripts in MDA-MB-231 and HCC1143 cells grown as monolayer or mammospheres. The experiment was performed in duplicate and the results are presented as mean ± SD. PureLink RNA mini kit (Life Technologies, Grand Island, NY, USA) was used to extract RNA and 20 ng RNA was used for reverse transcription using iScript cDNA Synthesis Kit (Bio-Rad Laboratories, Hercules,

CA, USA). The duplex TaqMan Gene Expression Assays for aquaporin-1 (AQP1; Hs01028916_m1; Applied Biosystems, Foster City, CA, USA) and the reference genes were CCSER2 (HS00982799_mH, Applied Biosystems, Foster City, CA, USA). Protein expression of (**b**) AQP1 and (**c**) activation of FAK in MDA-MB-231 and HCC1143 mammospheres exposed to VC (vehicle control) or C3. Total cell lysates were prepared and quantified. The experiments were repeated 2 times with 20 or 50 ug proteins. The antibodies used for immunostaining include anti-AQP1 antibody [EPR20325] (ab219055, Abcam, Cambridge, UK, 1:1000), anti-FAK antibody [EP6954] (ab40794, Abcam, Cambridge, UK, 1:1000), anti-phospho FAK antibody [EP2]60Y] phosphor Y397 (ab 81298, Abcam, Cambridge, UK, 1:1000). Goat anti-rabbit IgG H&L (HRP) (ab6721, Abcam, Cambridge, UK, 1:3000) was used as the secondary antibody.

## References


1. Palethorpe, H.M.; Smith, E.; Tomita, Y.; Nakhjavani, M.; Yool, A.J.; Price, T.J.; Young, J.P.; Townsend, A.R.; Hardingham, J.E. Bacopasides I and II act in synergy to inhibit the growth, migration and invasion of breast cancer cell lines. *Molecules* **2019**, *24*, 3539, doi:10.3390/molecules24193539.
2. Tomita, Y.; Palethorpe, H.M.; Smith, E.; Nakhjavani, M.; Townsend, A.R.; Price, T.J.; Yool, A.J.; Hardingham, J.E. Bumetanide-derived aquaporin 1 inhibitors, AqB013 and AqB050 inhibit tube formation of endothelial cells through induction of apoptosis and impaired migration in vitro. *Int. J. Mol. Sci.* **2019**, *20*, 1818.
